# Construction of a ceRNA Network and Analysis of Tumor Immune Infiltration in Pancreatic Adenocarcinoma

**DOI:** 10.3389/fmolb.2021.745409

**Published:** 2021-10-25

**Authors:** Jingjing Xiao, Chao Lv, Chuan Xiao, Jinyu Ma, Jun Liao, Tao Liu, Jun Du, Shi Zuo, Haiyang Li, Huajian Gu

**Affiliations:** ^1^ School of Clinical Medicine, Guizhou Medical University, Guiyang, China; ^2^ Department of Hepatobiliary Surgery, Guizhou Provincial People’s Hospital, Guiyang, China; ^3^ Department of Pediatric Surgery, The Affiliated Hospital of Guizhou Medical University, Guiyang, China; ^4^ Department of Hepatobiliary Surgery, The Affiliated Hospital of Guizhou Medical University, Guiyang, China

**Keywords:** Pancreatic adenocarcinoma, competing endogenous RNA network, tumor-infiltrating immune cell, prognostic model, TCGA

## Abstract

Pancreatic adenocarcinoma (PAAD) is characterized by high malignancy, frequent metastasis, and recurrence with an unfavorable prognosis. This study is aimed at constructing a prognostic model for tumor-infiltrating immune cells and a competing endogenous RNA (ceRNA) network in PAAD and analyzing susceptibilities of chemotherapy and immunotherapy of PAAD. Gene expression profiles and clinical information of PAAD were downloaded from The Cancer Genome Atlas (TCGA) database and divided into the tumor group and the normal group. A total of five PAAD survival-related key genes in the ceRNA network and three survival-related immune infiltrating cells were uncovered, and two survival risk models and nomograms were constructed. The efficiency and performance of the two models were verified using multi-index area under the curve analysis at different time points, decision curve analysis, and calibration curves. Co-expression analysis showed that LRRC1, MIR600HG, and RNF166 in the ceRNA network and tumor-infiltrating immune cells including CD8 T cells and M1 macrophages were likely related to the PAAD prognosis, and the expression of key ceRNA-related genes was experimently validated in tissues and cell lines by RT-qPCR. Patients with low risk scores for key genes in the ceRNA network displayed a positive response to anti-programmed death-1 (PD-1) treatment and greater sensitivity to chemotherapeutic drugs such as docetaxel, lapatinib, and paclitaxel. More importantly, our results suggested that the IC50 values of gemcitabine in PAAD were not significantly different between the high and low risk groups. The expression levels of immune checkpoints were significantly different in the high-risk and low-risk groups. The prognostic model, nomogram, and drug analysis may provide an essential reference for PAAD patient management in the clinic.

## Introduction

Pancreatic adenocarcinoma (PAAD) is a highly malignant gastrointestinal tumor with a highly aggressive metastatic capacity, and its five-year survival rate is less than 10% (Siegel et al., 2021). Tumors in approximately 53% of patients have metastasized at the time of diagnosis. The five-year survival rate for this group of patients is only 2.4% (Chen et al., 2020a). In 2020, 9.96 million worldwide had lost their lives due to cancer, of which 4,70,000 deaths were attributed to PAAD (GBD 2017 Disease and Injury Incidence and Prevalence Collaborators, 2018). PAAD is the fourth leading cause of death from malignant tumors and is expected to rank second globally by 2030 (Rahib et al., 2014; Qiao et al., 2019). Generally, early metastasis and drug resistance of PAAD are considered important reasons for its high mortality (Zhang et al., 2019; Chen et al., 2020a; Kong et al., 2020; Yang and Leung, 2020). Studies show that the infiltration pattern and activation of immune cells in various tumors are closely related to tumor progression and prognosis (Chen et al., 2020b; Shi et al., 2020; Wang et al., 2020). Although the research on PAAD has advanced greatly in terms of early diagnosis, surgical technique, and drug treatment in recent years, the prognosis of PAAD remains poor (Rawla et al., 2019; Yao et al., 2019; Zhang et al., 2019). Reducing the mortality of PAAD patients and improving their early diagnosis and prognosis urgently require understanding the effects of early-stage gene expression and immune cell infiltration on prognosis (Chou et al., 2016; Guo et al., 2020).

PAAD is considered to be resistant to immunotherapy (Park et al., 2019; Yoo et al., 2019). There are several hypotheses about the underlying mechanism, including the lack of effector cells in the tumor microenvironment accompanied by suppression of immune infiltration, the migration of impaired effector cells by dense matrix, and immune checkpoint signal transmission (Kesh et al., 2020). The progression of cancer is closely related to the tumor microenvironment. Studies have found that the composition and function of tumor-infiltrating immune cells vary with the host’s immune status and correlate with clinical features (Shibutani et al., 2018; Udall et al., 2018). There are complex interactions between tumor-infiltrating immune cells (TIICs) and tumor cells. Among them, some can support immune defense, while some promote tumor progression, thereby affecting prognosis (Mantovani and Sica, 2010).

In 2011, Salmena et al. (2011) proposed a competing endogenous RNA (ceRNA) hypothesis that tumor biological processes could be influenced by regulating the expression of target genes and microRNAs (miRNAs). Numerous pieces of studies also demonstrate that the ceRNA network can predict the prognosis of patients with PAAD (Wang et al., 2019; Xiong et al., 2019). However, simultaneous analysis of ceRNA networks and immune cell infiltration patterns to predict clinical outcomes of patients with PAAD has not been performed. Therefore, we used The Cancer Genome Atlas (TCGA) database to construct the PAAD ceRNA network and evaluated the prognostic value of tumor immune cell infiltration through bioinformatics tools.

Herein, the expression profile data of PAAD samples in TCGA database were adopted to establish a ceRNA network related to the prognosis of PAAD. Furthermore, the immune cell composition was calculated with CIBERSORT, and immune cells related to the prognosis of PAAD were analyzed. A PAAD prognostic risk model was established based on important immune cells and key genes in the ceRNA network, and its predictive efficiency was verified in multiple dimensions. Next, drug analysis was performed on the ceRNA network in the high-risk and low-risk groups. The constructed nomogram is expected to provide novel insights into the prognosis and targeted therapy of PAAD.

## Materials and Methods

### Tissue Specimen Collection

A total of 12 PAAD tissues and the corresponding paracancerous tissues were collected from the Affiliated Hospital of Guizhou Medical University from August 2020 to April 2021. This study was approved by the Ethics Committee of Guizhou Medical University, and all participants with PAAD provided informed consent forms.

### Cell Culture

PAAD cell lines PANC-1, MIA PaCa-2, BxPC-3, AsPC-1, and SW1990 were purchased from the American Type Culture Collection (Manassas, VA, United States), and the normal human pancreatic duct epithelial (HPDE) cell line was purchased from Beijing North Carolina Chuanglian Biotechnology Research Institute (Beijing, China). All cell lines were cultured in the corresponding medium with 10% fetal bovine serum (Gibco, Waltham, MA, United States).

### Raw Data Collection and Differential Expression Analysis

Gene expression profiles of 182 PAAD patients (178 tumor samples and 4 normal samples) were downloaded from TCGA (https://portal.gdc.cancer.gov/) database, including lncRNAs, mRNAs, miRNAs, HTseq (a python package) counts, fragments per kilobase of exon per million mapped fragments (FPKM), and the clinical information of each patient. Then, the PAAD group and the normal group were filtered through Perl language, the differentially expressed lncRNAs, mRNAs, and miRNAs were identified using the “DEseq2” package in R language (version 4.0.2), and heatmaps and volcano maps were plotted. DEG’s filtering criteria were as follows: fold change (FC) of differential expression of lncRNAs and mRNAs was logFC > 2, −logFC < −1 (miRNA) and *p value* < 0.01.

### Construction and Functional Annotation of the ceRNA Network

The correlation analysis of differentially expressed lncRNAs and mRNAs in TCGA database was performed with the “GDCRNATools” package in R language. Then, the differentially expressed miRNAs that can regulate lncRNAs and mRNAs were identified using the hypergeometric test and correlation analysis to construct a ceRNA network (*p* < 0.05 as the filter threshold criteria). The PAAD ceRNA network was visualized by introducing edge and nodal gene information into the interaction network with Cytoscape v3.8.0. Subsequently, differentially expressed genes (DEGs) in the ceRNA network were annotated with Gene Ontology (GO) and Kyoto Encyclopedia of Genes and Genomes (KEGG). The above analysis was performed with the “clusterProfiler,” “org.Hs.eg.db,” “enrichplot,” and “ggplot2” packages in R language, and then Cytoscape was employed for the GO/KEGG network analysis. Among them, GO analysis was utilized for identifying the biological characteristics, including biological processes (BPs), cellular components (CCs), and molecular functions (MFs). Meanwhile, KEGG analysis was used to explore the biological pathways.

### Construction of a Prognostic Model of Key Genes in the ceRNA Network

Based on the clinical information of PAAD patients downloaded from TCGA database, the survival time and state were extracted. Kaplan–Meier survival analysis and univariate Cox regression analysis were conducted by integrating clinical data and ceRNA network node information to evaluate the prognostic value of node genes. Lasso regression (conducted via the “glmnet” package in R language) was used to remove redundancy factors to prevent over-fitting of the model, and then the ceRNA network prognosis model was obtained through multivariate Cox regression analysis.

### Evaluation of the Prognostic Model of Key Genes in the ceRNA Network and Construction of the Nomogram

All patients were divided into high-risk and low-risk groups according to the median of the model’s risk score, and the survival curve was drawn to compare the differences in survival between them. The area under the curve (AUC) of the receiver-operating characteristic (ROC) curve (visualized by the “plotAUCcerve” package in R language) was used to evaluate the prediction efficiency of the model. Next, a decision curve analysis (DCA) curve was plotted with the “ggDCA” package of R, and a nomogram was constructed with the “rms” package of R. Furthermore, the calibration curve was used to evaluate the accuracy of the nomogram in predicting the survival rate of patients with PAAD.

### Analysis of Chemosensitivity and Immunotherapy of Key Genes in the ceRNA Network

The Genomics of Drug Sensitivity in Cancer (GDSC, https://www.cancerrxgene.org) (Yang et al., 2013), the largest available database, was used to evaluate the sample’s half-maximal inhibitory concentration (IC50) and predict the chemotherapy response of samples with the “pRRophetic” package of R. We selected eight common medicines (A.443654, BI.2536, lapatinib, dasatinib, gemcitabine, docetaxel, paclitaxel, and cyclopamine) to predict the IC50 value of each sample. Subsequently, the difference between the IC50 values in the high-risk and low-risk groups was analyzed using the Wilcoxon rank-sum test. Additionally, the Tumor Immune Dysfunction and Exclusion (TIDE) algorithm and subclass mapping were used to predict the immune responses of the sample to anti-PD-1 and anti-CTLA-4 treatment.

### CIBERSORT Estimation

The abundance of 22 immune cell subgroups in 178 tumor samples was calculated by the “CIBERSORT” package in R language, with *p* < 0.05 as the threshold. The “Pheatmap” package of R was used to visualize the infiltration of immune cells in the normal and tumor groups. The Wilcoxon rank-sum test was used to analyze the differentially expressed tumor-infiltrating immune cells in PAAD tissues and normal tissues. Then, Kaplan–Meier analysis was conducted to identify prognosis-related immune cells.

### Nomogram Construction and Evaluation of the Risk Prognosis Model of Tumor Immune Cells

We used Kaplan–Meier survival analysis and univariate Cox regression to investigate the survival-related immune cells. At the same time, the final immune cell model was obtained by Lasso regression and multivariate Cox regression analysis, and the model’s accuracy was evaluated by the AUC value. We used the multi-index AUC value and DCA to estimate the quantification of the prognostic model and utilized the calibration curve to assess the predictive accuracy of the nomogram. Meanwhile, we used Pearson correlation analysis to explore the relationship between key genes in the ceRNA network and immune cells in the model.

### Development of the Risk Model of Key Genes in the ceRNA Network

Hereby, we confirmed the tissue and cell expression levels of one lncRNA and three mRNAs in the model through experiments. In particular, TCGA and GTEx RNAseq data in TMP format that were extracted from the UCSC Xena browser (https://xenabrowser.net/datapages/) (Vivian et al., 2017) were uniformly processed using the Toil process. The PAAD data in TCGA and the corresponding normal tissue sample data in GTEx were extracted for differential expression analysis with the “EdgeR” package of R. Then, the expression levels of key genes were validated by real-time quantitative reverse transcription PCR (RT-qPCR) in PAAD tissues and cell lines.

### RT-qPCR

To extract total RNA from tissue, soybean-sized samples of PAAD tissues and paracancerous tissues were removed from liquid nitrogen and then immediately treated with Trizol (Invitrogen, Carlsbad, CA, United States). Then, RT-qPCR was applied using the cDNA Kit (Genstar, Beijing, China) and qPCR Kit (Genstar, Beijing, China) based on the manufacturer’s instruction. The relative mRNA expression was calculated with the 2^–ΔΔCT^ method. The primers are presented in Table 1.

**TABLE 1:** Sequences of gene-specific primers used for real-time RT-qPCR.

Gene	Primer sequence
MIR600HG, forward	5′-TGA​GCA​GAG​TCA​AGT​GGC​AG-3′
MIR600HG, reverse	5′-AAA​GCC​CCA​TTT​CCT​AGC​CC-3′
RNF166, forward	5′-ACC​TGC​CAA​GTA​TGA​TGA​CAT​CA-3′
RNF166, reverse	5′-GGT​CCT​CAG​TGT​AGC​CCA​AGA​T-3′
LRRC1, forward	5′-TCC​TTA​CCA​AAA​GAG​ATC​GG-3′
LRRC1, reverse	5′-GGT​AGA​TGC​AGC​AAC​CTG​T-3′
LY6D, forward	5′-AGA​CAG​CTC​TGC​TCG​TCC​TC-3′
LY6D, reverse	5′-CTG​TAG​TCG​GAG​GTG​CAT​GA-3′
GAPDH, forward	5′-CAA​TGA​CCC​CTT​CAT​TGA​CC-3′
GAPDH, reverse	5′-GAC​AAG​CTT​CCC​GTT​CTC​AG-3

### Statistical Analysis

**FIGURE 1:**
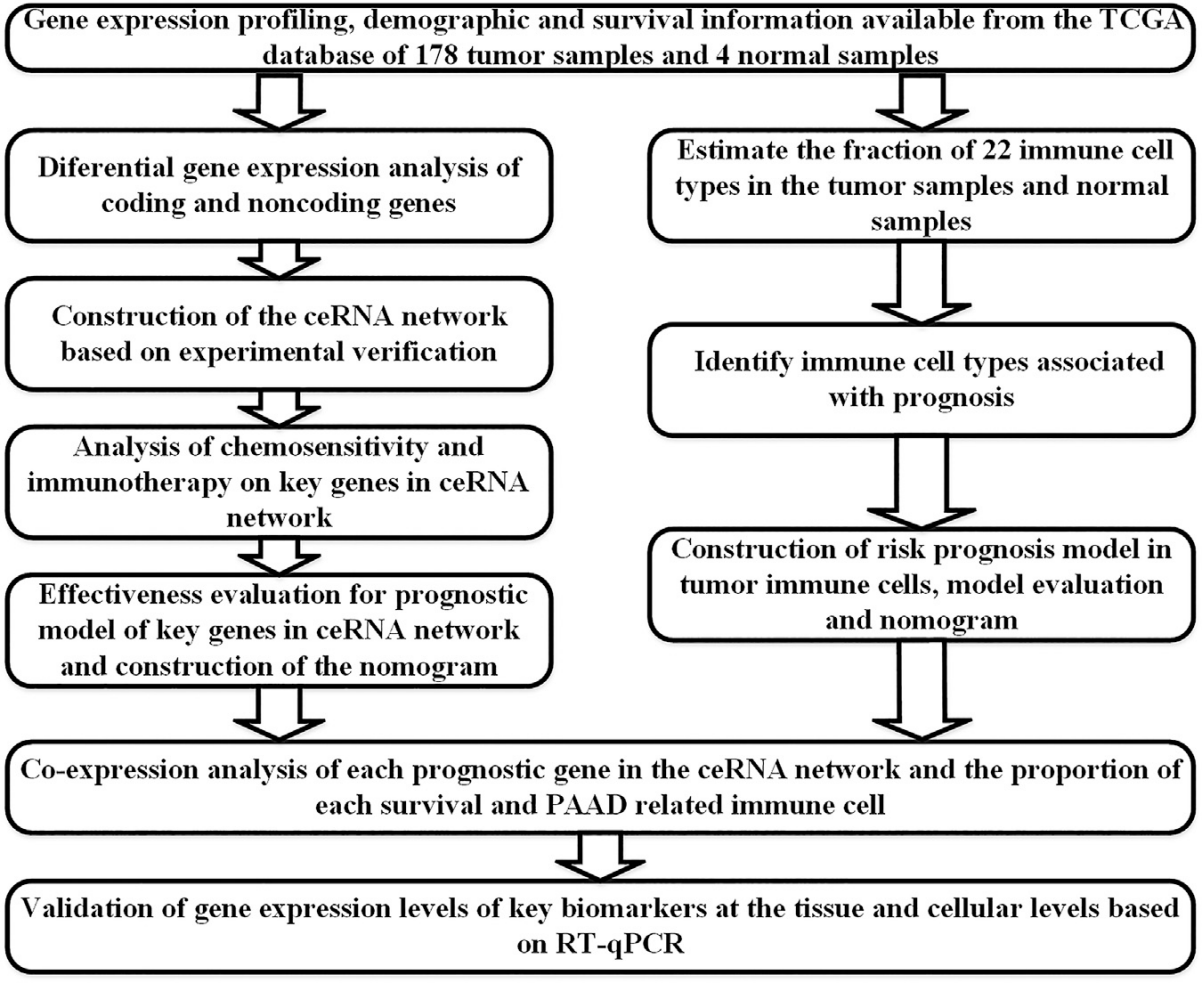
Flow chart of the analytical process.

All statistical analyses were performed with R (version 4.0.2). Bioconductor packages (Eivazi et al., 2016) used were as follows: limma, GDCRNATools, edgeR, ggplot2, survminer, timeROC, ggDCA, rms, glmnet, plotAUCcerve, and preprocessCore. Co-expression analysis was performed using the Pearson correlation analysis. The comparison of means was conducted with the independent sample Student’s t-test. GraphPad Prism 8 was used for the corresponding statistical analysis. *p values* < 0.05 on two sides were considered statistically significant. The flow chart of this study is shown in Figure 1.

## Results

### Identification of DEGs in PAAD in TCGA Database

**FIGURE 2:**
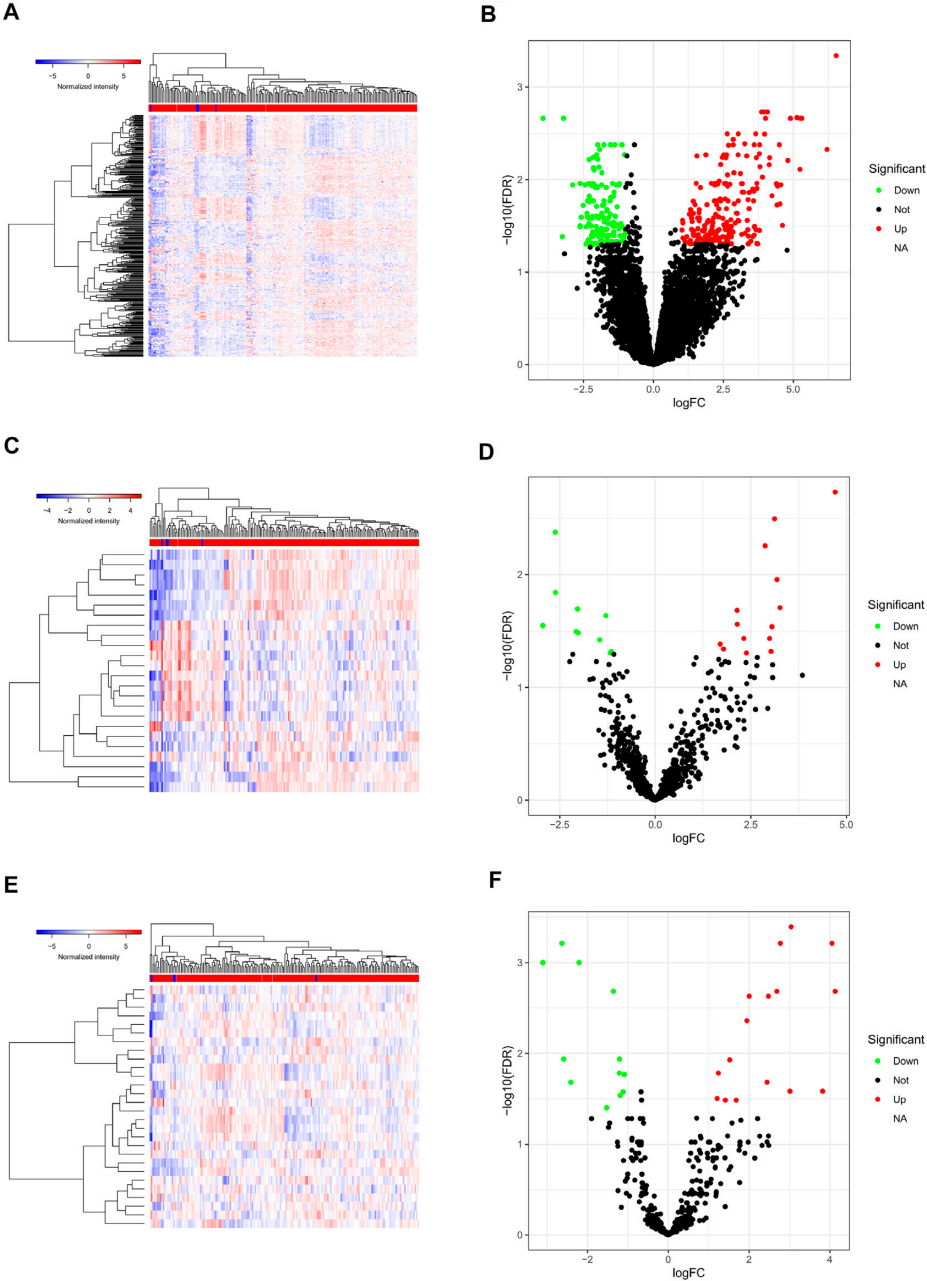
Differentially expressed genes in PAAD. Heatmap **(A)** and volcano plot **(B)** of all differentially expressed mRNAs; heatmap **(C)** and volcano plot **(D)** of all differentially expressed lncRNAs; heatmap **(E)** and volcano plot **(F)** of all differentially expressed miRNAs. PAAD, pancreatic adenocarcinoma; miRNAs, microRNAs; lncRNAs, long non-coding RNAs; mRNAs, messenger RNAs.

A total of 412 DEGs in PAAD were identified from TCGA database with the filter threshold of FC > 2 and false discovery rate (FDR) < 0.01, including 360 differentially expressed mRNAs (147 downregulated and 213 upregulated; Figures 2A,B), 28 differentially expressed miRNAs (16 upregulated and 12 downregulated; Figures 2C,D), and 24 differentially expressed lncRNAs (14 upregulated and 10 downregulated; Figures 2E,F).

### Construction and Functional Annotation of the ceRNA Network

**FIGURE 3:**
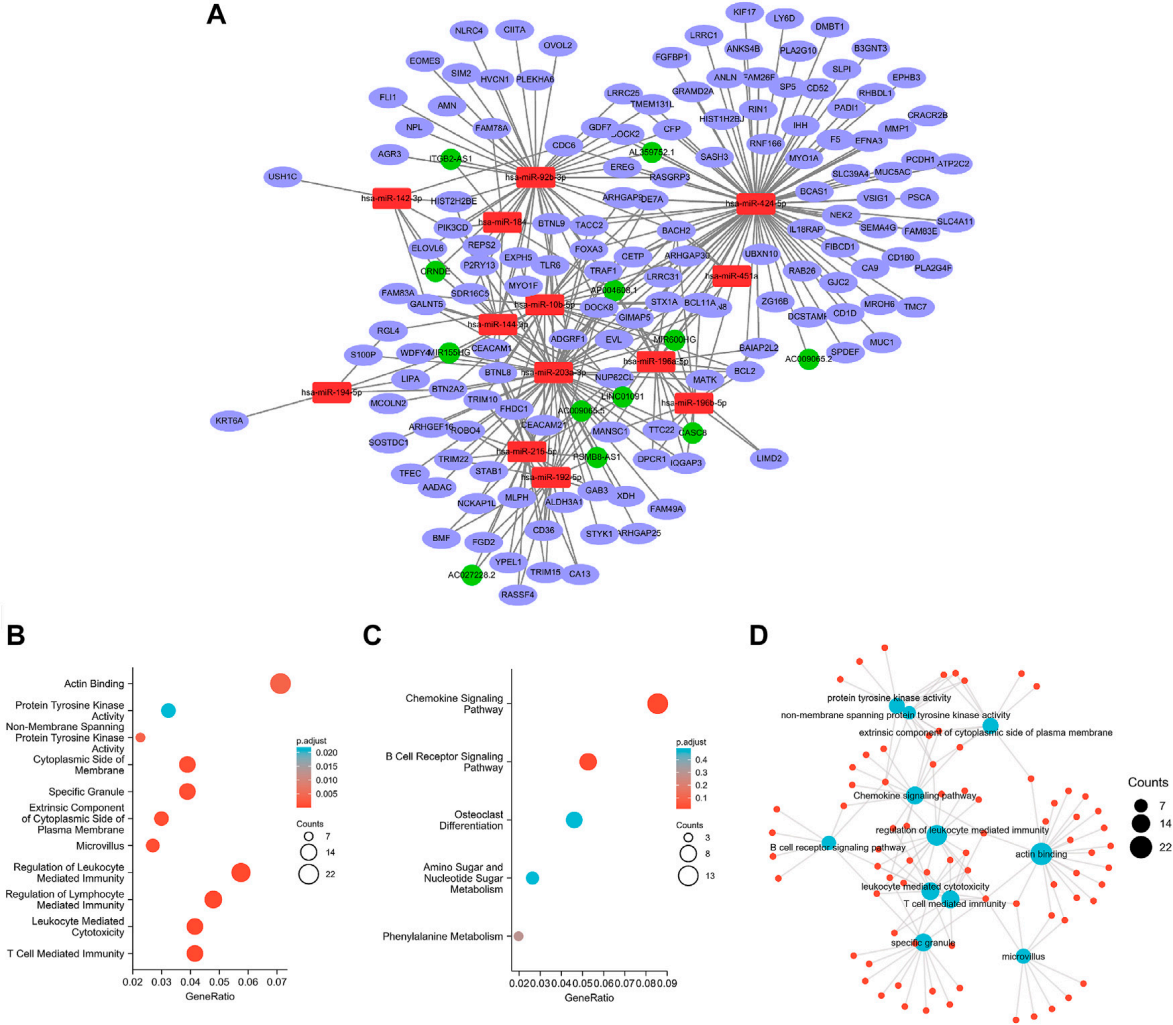
Constructed ceRNA networks via Cytoscape **(A)**; GO **(B)** and KEGG **(C)** analyses for ceRNA-related differentially expressed genes; visual network **(D)**.

After miRcode matching and miRNA target gene prediction, 12 differentially expressed lncRNAs, 13 miRNAs, and 145 mRNAs were identified. With 132 nodes and 311 edges, the ceRNA regulatory network of the PAAD-related DEGs for lncRNA, miRNA, and mRNA was constructed (Figure 3A). Function analysis of the KEGG signaling pathway and GO analysis (Figures 3B,C) revealed that the DEGs were mainly enriched in the integrin-mediated signaling pathway and chemokine signaling pathway, with a close relationship to regulated exocytosis, actin cytoskeleton organization, regulation of response to biotic stimulus, and regulation of leukocyte-mediated immunity. Furthermore, 11 significantly correlated GO and KEGG items were determined and visualized according to genes (CCs, MFs, BPs, and KEGG signaling pathway; Figure 3D).

### Nomogram Construction and Evaluation of the Prognostic Model of Key Genes in the ceRNA Network

**FIGURE 4:**
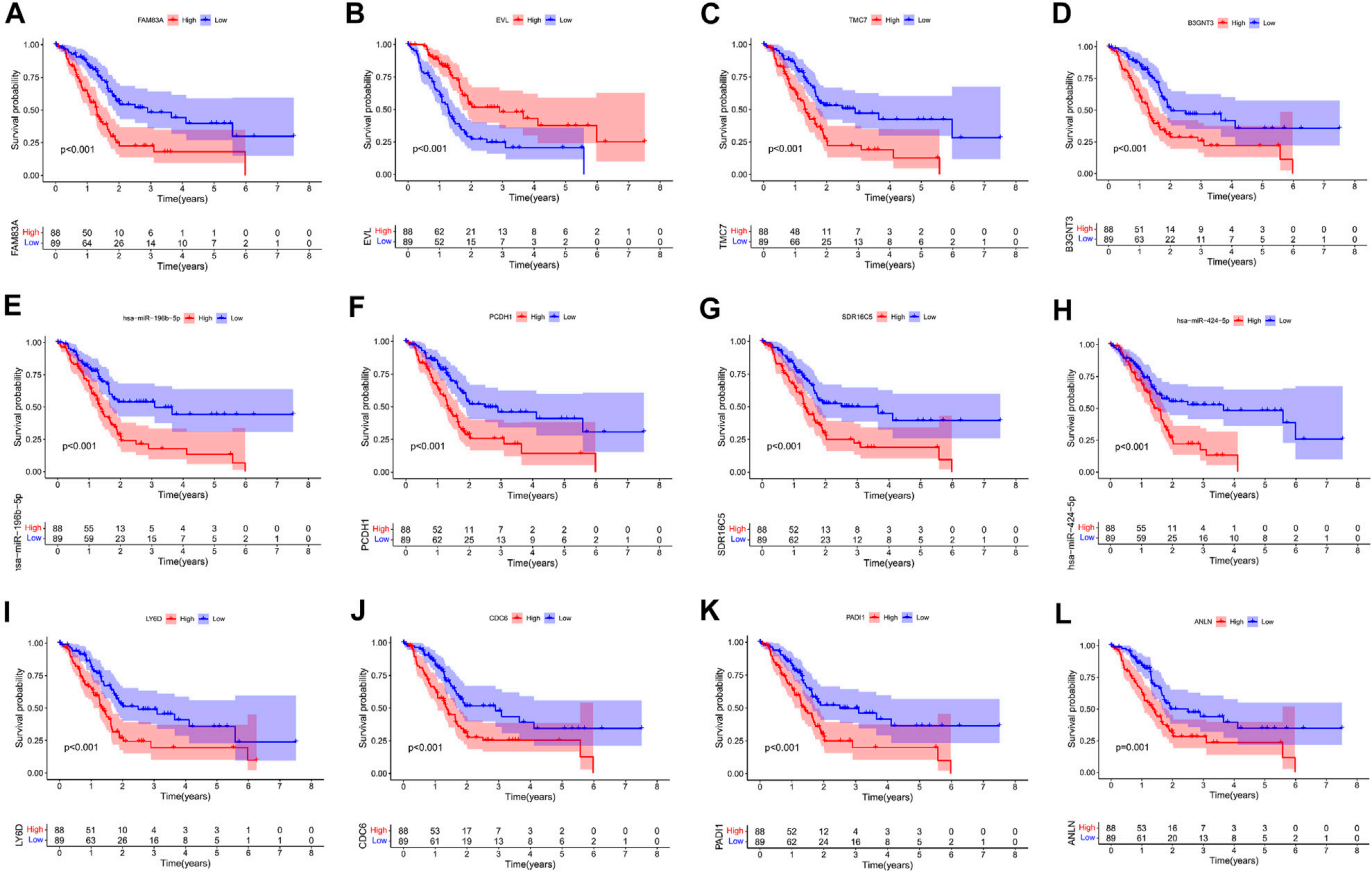
Kaplan–Meier survival curves of the former 12 genes in the ceRNA networks: FAM83A **(A)**, EVL **(B)**, TMC7 **(C)**, B3GNT3 **(D)**, hsa-miR-196b-5p **(E)**, PCDH1 **(F)**, SDR16C5 **(G)**, hsa-miR-424-5p **(H)**, LY6D **(I)**, CDC6 **(J)**, PADI1 **(K)**, and ANLN **(L)**.

**FIGURE 5:**
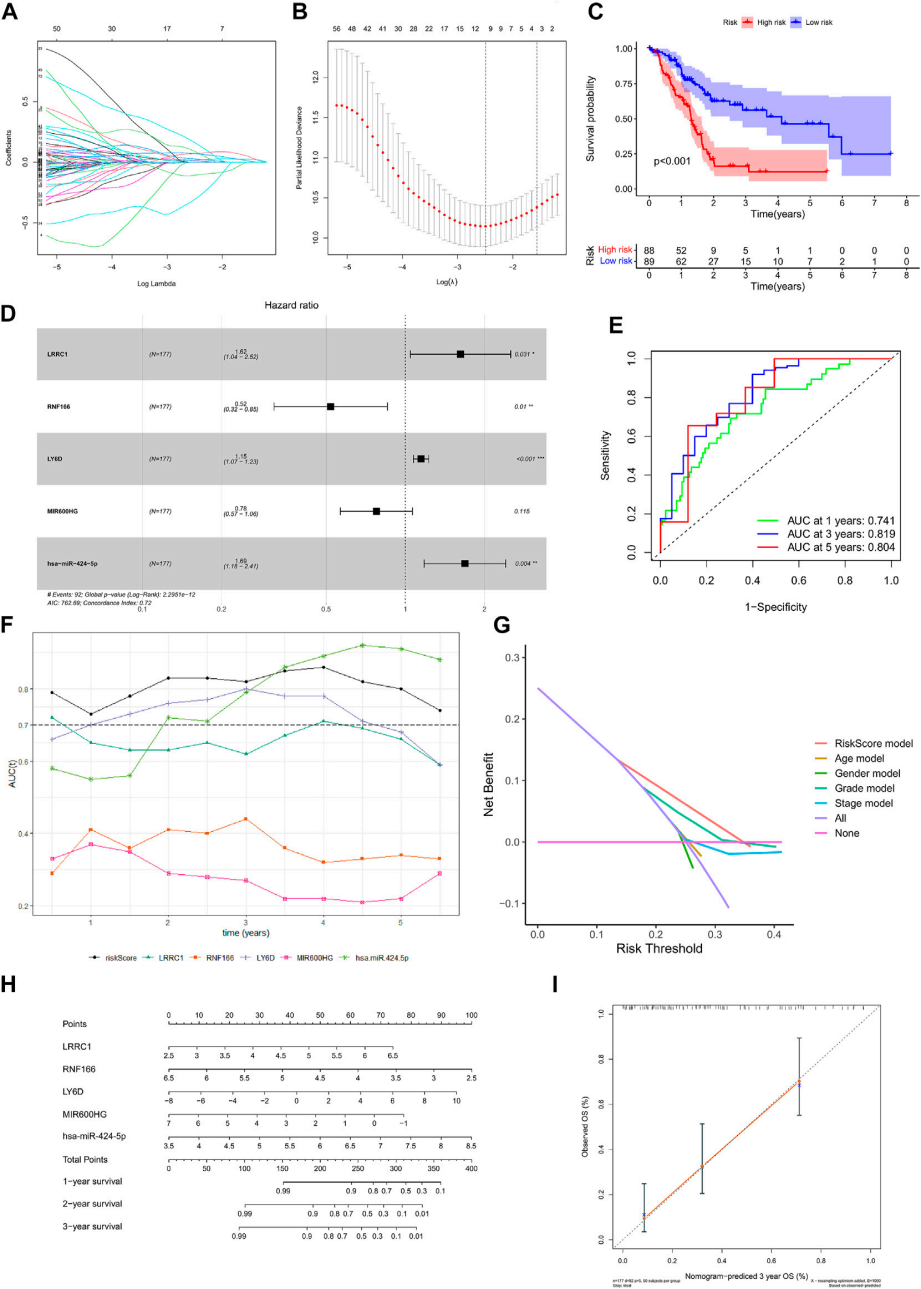
Construction of the nomogram for predicting the survival probability of PAAD based on the identified hub genes in ceRNA networks. Results of Lasso regression **(A, B)**; Kaplan–Meier survival curve in high- and low-risk groups based on the multivariate Cox regression analysis **(C)**; forest plot of the multivariate Cox regression analysis **(D)**; ROC curves of the multivariate Cox model **(E)**; area under the multi-indicator ROC curve **(F)** and decision-making curve **(G)**; nomogram **(H)** and its calibration curve **(I)**.

Kaplan–Meier survival analysis and log-rank tests were used to determine the survival difference of genes in the ceRNA network, which identified 41 genes correlated with PAAD prognosis. Then, the top 12 genes were filtered according to their *p* value with the log-rank test, and the Kaplan–Meier survival curve was plotted (Figure 4). Key genes in the network were further identified by univariate Cox regression analysis and Lasso regression analysis. We found that nine significant genes were included in the multivariate Cox regression analysis (Figures 5A,B). Next, through multivariate Cox regression analysis, five key genes in the ceRNA network were used to establish the prognostic model of PAAD, namely, LRRC1, RNF166, LY6D, MIR600HG, and hsa-miR-424-5p (Figure 5D and Table 3). The results of Kaplan–Meier curves indicated that patients in the high-risk group obtained a poorer prognosis than those in the low-risk group (Figure 5C). Meanwhile, the AUC of the ROC curve was generated to assess the predictive accuracy of the Cox regression risk model. Next, we found that its accuracy was great (the AUC values for one-, three-, and five-year survival were 0.741, 0.819, and 0.804, respectively) (Figure 5E). The comparison of multi-index AUC values at multiple time points revealed that the prediction accuracy of the early-stage model was better than that of the individual genes, with hsa-miR-424-5p at the end stage showing better prediction (Figure 5F). The DCA indicated that the risk model had better prediction performance than the model constructed by traditional clinicopathological features (Figure 5G). The final nomogram was constructed by the expression of key genes in the model, and the calibration curve illustrated the higher predictive accuracy of the nomogram (Figures 5H,I).

### Analysis of Chemosensitivity and Immunotherapy in the ceRNA Network Prognostic Model

**FIGURE 6:**
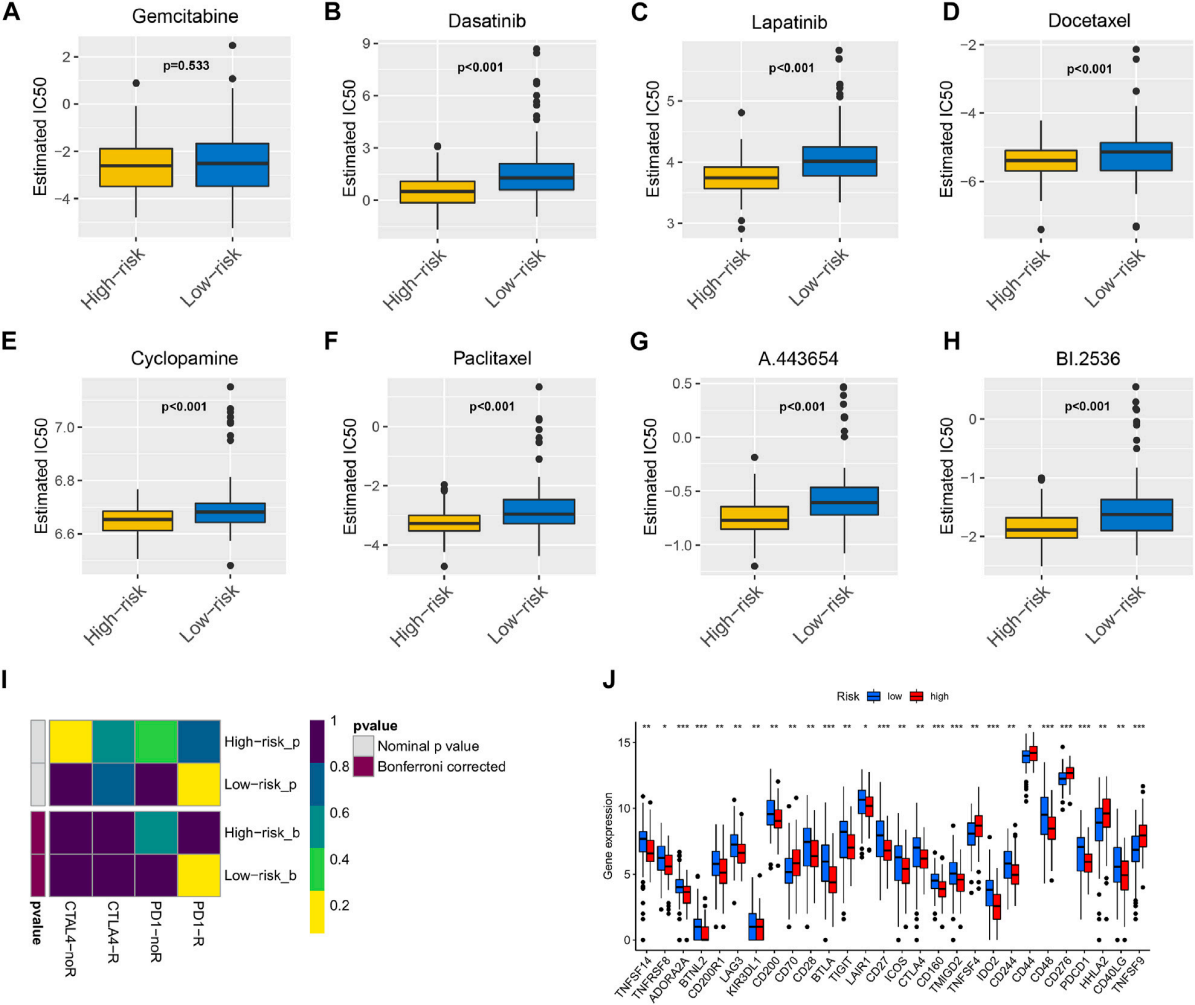
The model acted as a potential predictor of chemosensitivity as high risk scores were related to a lower IC50 for chemotherapeutics such as doxorubicin, mitomycin, and cisplatin, whereas they were related to a higher IC50 for gemcitabine Box plots depicted the differences in the estimated IC50 levels of **(A)** Gemcitabine; **(B)** Dasatinib; **(C)** Lapatinib; **(D)** Docetaxel; **(E)** Cyclopamine; **(F)** Paclitaxel; **(G)** A.443654; **(H)** BI.2536 between the high and low risk score groups. The results illustrated that the group with low risk score was more likely to respond to immune checkpoint inhibitors (PD-1 and CTLA-4 inhibitors) than the group with high risk score **(I)**; expression of immune checkpoints among high and low PAAD risk groups **(J)**.

The chemotherapeutics gemcitabine (Figure 6A), dasatinib (Figure 6B), lapatinib (Figure 6C), docetaxel (Figure 6D), cyclopamine (Figure 6E), paclitaxel (Figure 6F), A.443654 (Figure 6G), and BI.2536 (Figure 6H) were compared in the high-risk and low-risk groups. We found that gemcitabine had no difference between the high-risk and low-risk groups, and the remaining seven drugs showed higher sensitivity in the high-risk group than the low-risk group. As shown in Figure 6I, low-risk patients had a significantly positive response to anti-PD-1 treatment. Differential analysis of immune checkpoint expression revealed that the expression levels of immune checkpoint factors such as PD-1, CTLA-4, LAG3, and BTLA were significantly different between the high-risk and low-risk groups (Figure 6J).

### Nomogram Construction and Evaluation of Prognostic Risk Model of TIICs

**FIGURE 7:**
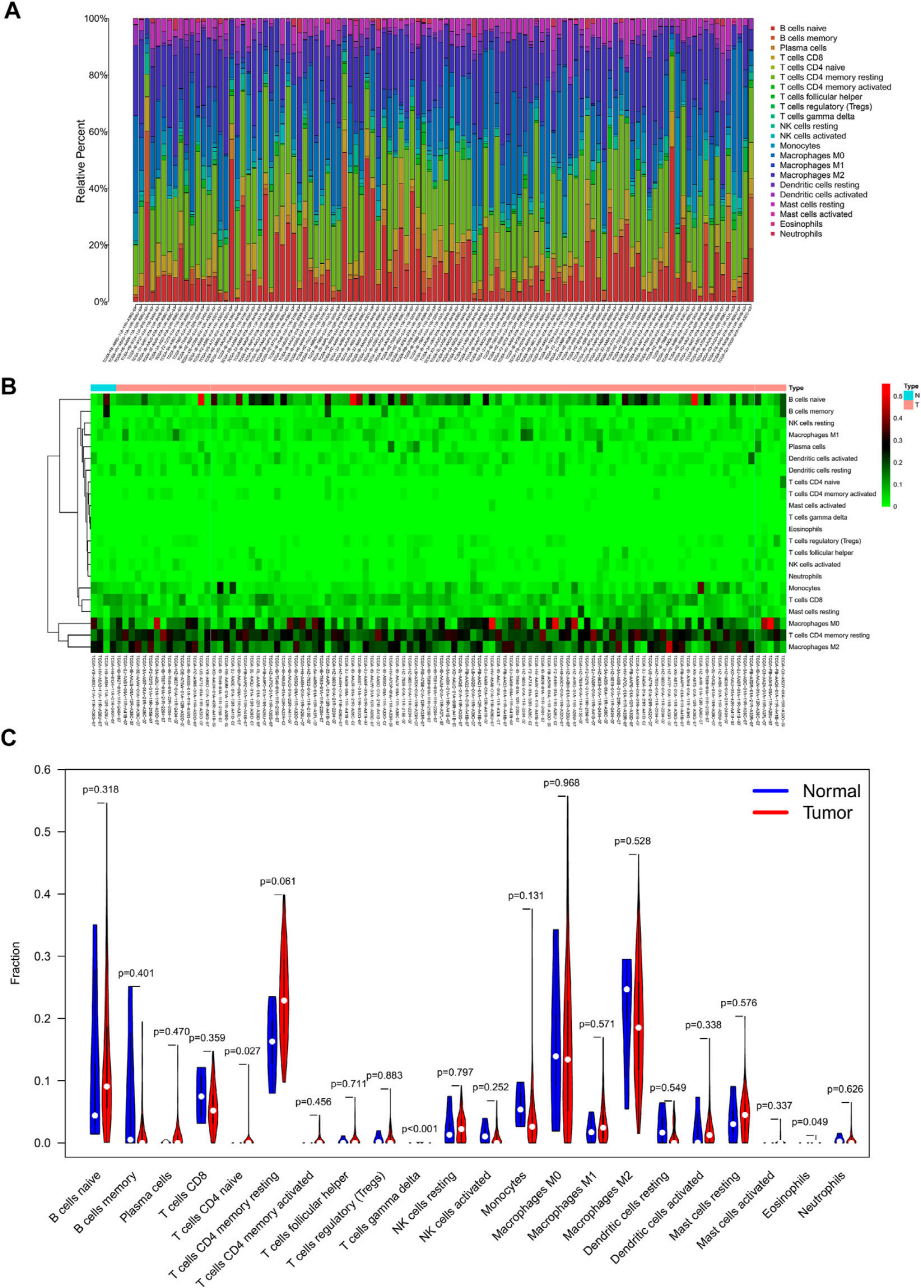
Composition **(A)** and heatmap **(B)** of 22 subsets of immune cells in PAAD; violin plot **(C)** of immune cell infiltration in tumor and normal groups.

**FIGURE 8:**
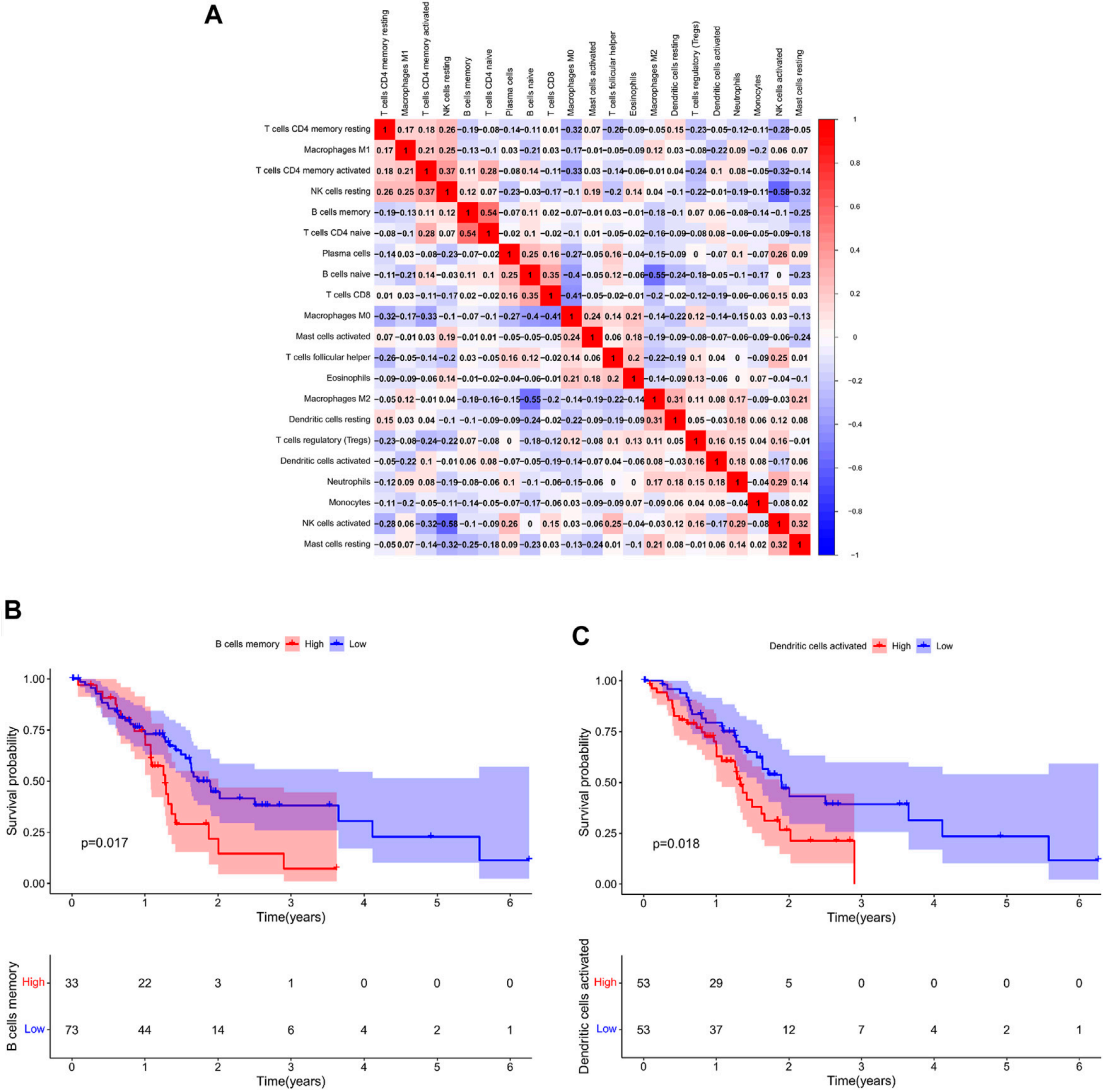
**(A)**: Co-expression patterns among fractions of immune cells; Kaplan–Meier survival curves of fractions of memory B cells **(B)** and activated dendritic cells **(C)**.

**FIGURE 9:**
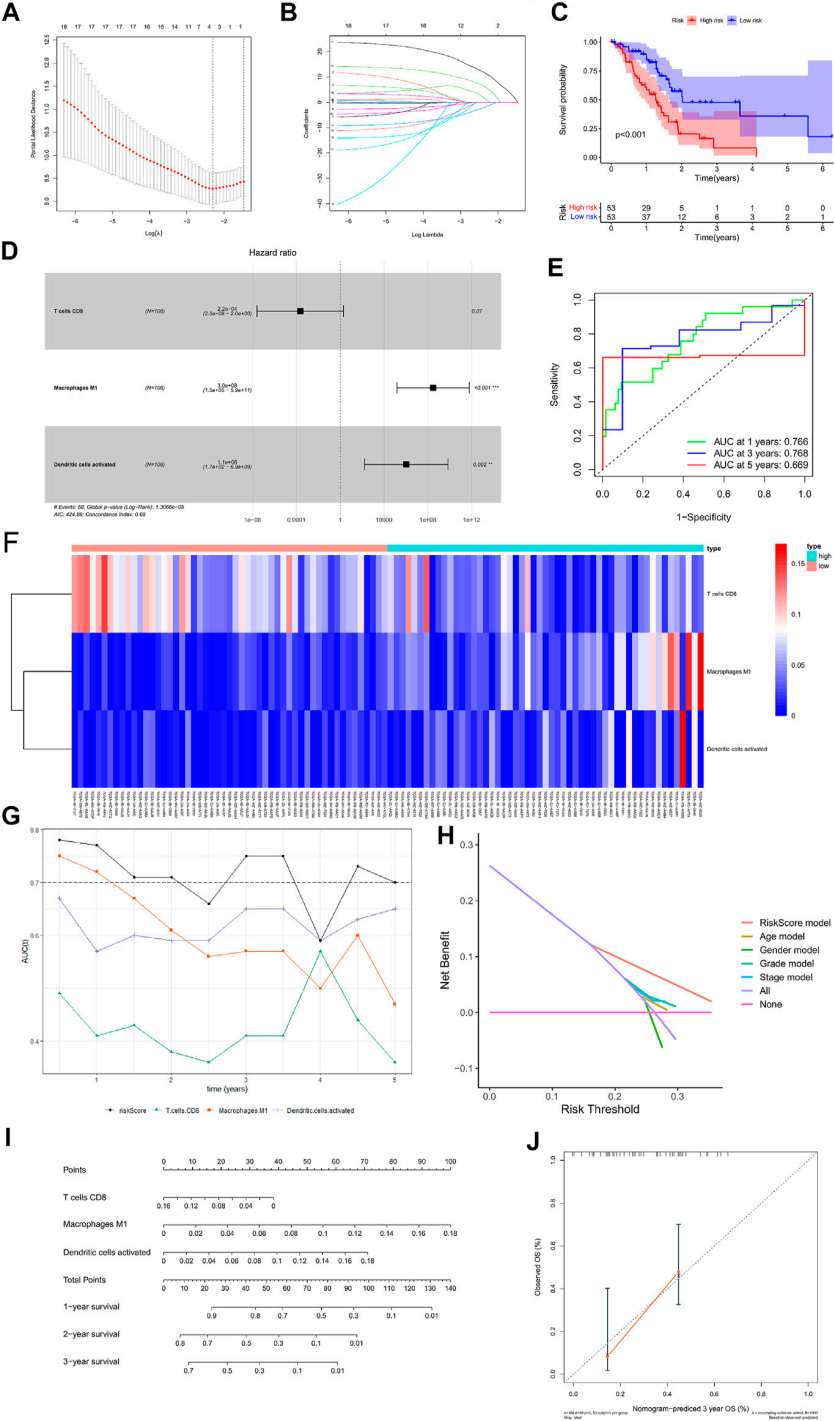
Construction of the nomogram for predicting the survival probability of PAAD based on the prognostic-related immune cells. Results of Lasso regression **(A, B)**; Kaplan–Meier survival curve in high- and low-risk groups based on the multivariate Cox regression analysis **(C)**; forest plot of the multivariate Cox regression analysis **(D)**; ROC curves of the multivariate Cox model **(E)**; heatmap of the three immune cells in the Cox regression model **(F)**; area under the multi-indicator ROC curve **(G)** and decision-making curve **(H)**; nomogram **(I)**; nomogram-predicted probability of three-year overall survival **(J)**.

The percentage of 22 immune cells in all samples was obtained using the deconvolution algorithm CIBERSORT, and a histogram and heatmap of the immune cells were drawn (Figures 7A,B). We found that naive CD4 T cells, resting memory CD4 T cells, eosinophils, and gamma delta T cells were significantly infiltrated in PAAD samples, whereas memory B cells, memory monocyte B cells, and resting dendritic cells were significantly infiltrated in normal tissue samples (Figure 7C). Moreover, Pearson correlation analysis suggested that naive CD4 T cells were significantly positively correlated with memory B cells (*r* = 0.58), and activated NK cells were significantly negatively correlated with resting NK cells (*r* = −0.58) (Figure 8A). The PAAD prognosis-related immune cells were identified by Kaplan–Meier survival analysis and log-rank tests, which showed that the infiltration levels of memory B cells and activated dendritic cells in tumor tissues were significantly correlated with PAAD prognosis (Figures 8B,C). Subsequently, under Lasso regression (Figures 9A,B) and Cox regression analysis (Figure 9D, Table 2), CD8 T cells, M1 macrophages, and activated dendritic cells were incorporated into the model. The results of Kaplan–Meier curves demonstrated that patients in the high-risk group obtained a poorer prognosis than those in the low-risk group (Figure 9C). The levels of CD8 T cell, M1 macrophage, and activated dendritic cell infiltration in the high-risk and low-risk groups are shown via a heatmap (Figure 9F). The AUC of the ROC curve was generated to assess the predictive accuracy of the Cox regression risk model, which was found to be excellent (the AUC values for one-, three-, and five-year survival were 0.766, 0.768, and 0.669, respectively) (Figure 9E). The comparison of the multi-index AUC values at multiple time points revealed that the predictive efficacy of the model was significantly better than that of individual immune cells at all time points (Figure 9G). The DCA indicated that the immune risk model had better predictive performance than the model constructed by traditional clinicopathological features (Figure 9H). The final nomogram was constructed based on the content of immune cells in the model, and the calibration curve illustrated the high accuracy of the nomogram in predicting survival (Figures 9I,J).

**TABLE 2:** Multivariate Cox proportional hazards regression model including the key immune cells for overall survival in patients with PAAD.

Gene	Coefficient	HR	95% CI		*p* value
			Lower	Upper	
CD8 T cells	−8.41	0.000	2.50E−08	1.20	0.070
M1 macrophages	19.53	302,109,492.5	153,468.35	5.94716E+11	4.52E−07
Activated dendritic cells	13.89	1,075,653.89	166.67	6.94E+09	0.002

PAAD, pancreatic adenocarcinoma; HR, hazards ratio; CI, confidence interval.

**TABLE 3:** Multivariate Cox proportional hazards regression model including the key members of the ceRNA network for overall survival in patients with PAAD.

Gene	Coefficient	HR	95% CI		*p* value
			Lower	Upper	
LRRC1	0.48	1.62	1.04	2.52	0.031
RNF166	−0.65	0.52	0.32	0.85	0.009
LY6D	0.14	1.15	1.07	1.23	0.001
MIR600HG	−0.25	0.78	0.57	1.06	0.115
hsa-miR-424-5p	0.52	1.69	1.18	2.41	0.004

PAAD, pancreatic adenocarcinoma; HR, hazards ratio; CI, confidence interval.

### Co-Expression Analysis

**FIGURE 10:**
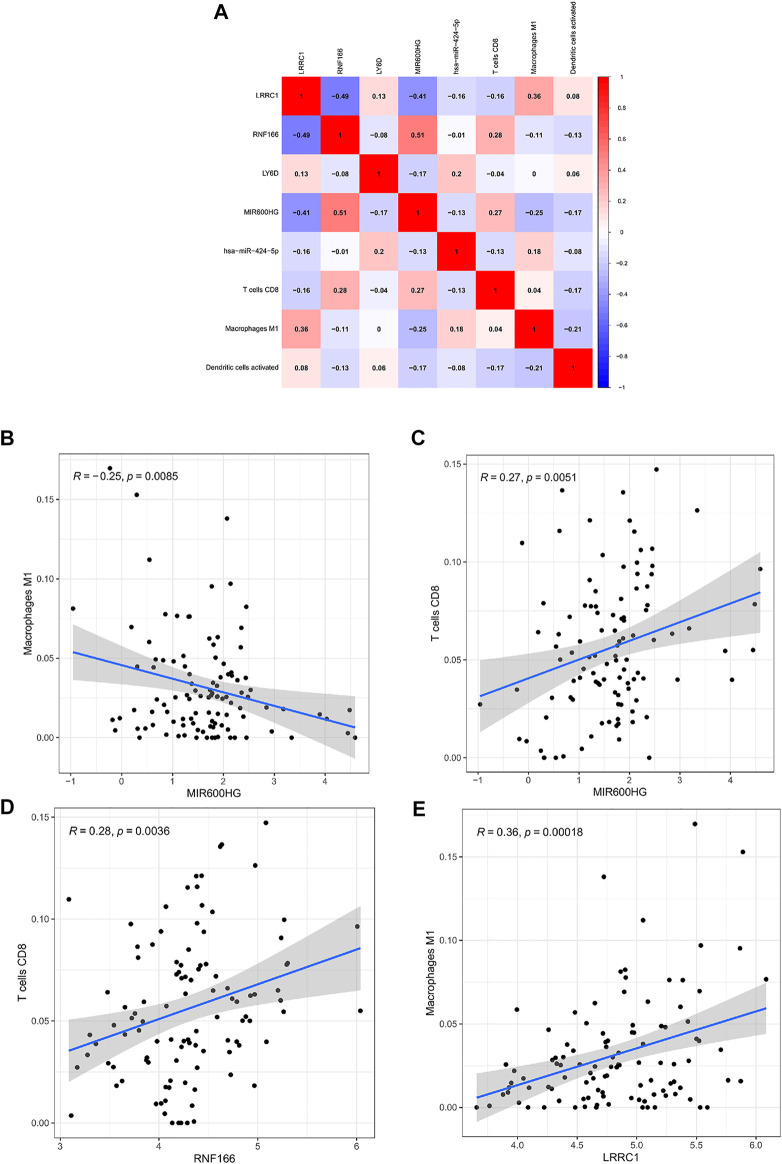
Co-expression patterns among fractions of three immune cells and five hub genes in the ceRNA network **(A)**; MIR600HG and M1 macrophages **(B)**; MIR600HG and CD8 T cells **(C)**; RNF166 and CD8 T cells **(D)**; LRRC1 and M1 macrophages **(E)**.

Co-expression analysis was conducted through the Pearson correlation test to explore the correlation between key genes in the ceRNA network and prognosis-related immune cells (Figure 10A). We found that MIR600HG and M1 macrophages were inversely correlated (*R* = −0.250; *p* < 0.01) (Figure 10B), MIR600HG and CD8 T cells were positively correlated (*R* = 0.270; *p* < 0.01) (Figure 10C), RNF166 and CD8 T cells were positively correlated (*R* = 0.280; *p* < 0.01) (Figure 10D), and LRRC1 and M1 macrophages were significantly positively correlated (*R* = 0.355; *p* < 0.001) (Figure 10E).

### Experimental Validation of the Expression Levels of Key Genes in the ceRNA Network

**FIGURE 11:**
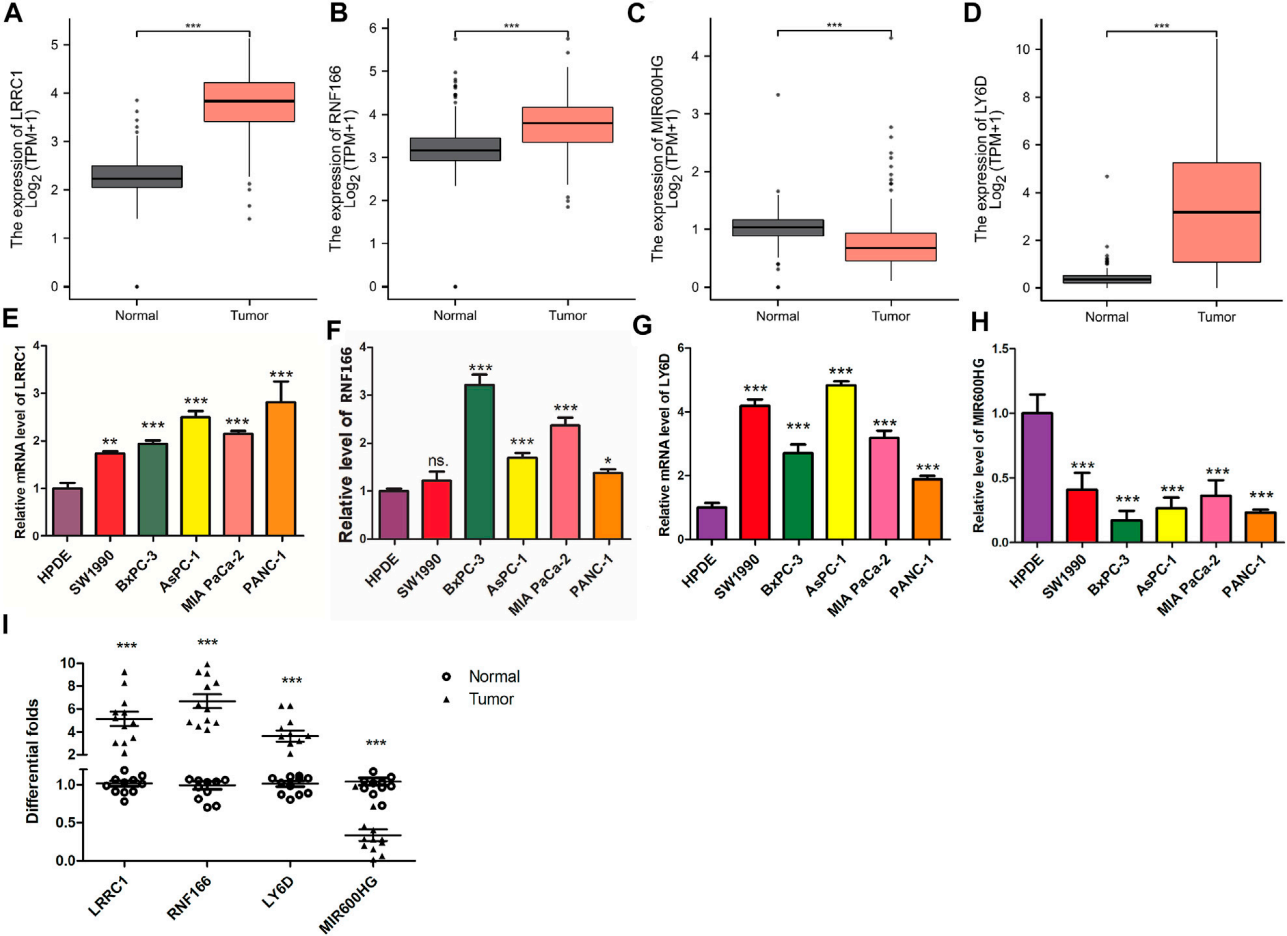
Validation of the expression levels of the four hub genes between normal pancreatic samples and PAAD samples based on TCGA and GTEx data in GEPIA. Expression levels of LRRC1, RNF166, LY6D, and MIR600HG in normal pancreatic samples and PAAD samples **(A–D)**. RT-qPCR was applied to test LRRC1 **(E)**, RNF166 **(F)**, LY6D **(G)**, and MIR600HG **(H)** expressions in PC cell lines.Validation of the expression levels of the four hub genes between normal pancreatic samples (*n* = 12) and PAAD samples (*n* = 12) by PCR analysis **(I)**. All of the data are presented as mean ± SD. Significant differences are defined by a *p* value < 0.01.

Compared to normal tissues, we found that the expression levels of LRRC1, RNF166, and LY6D were significantly higher in PAAD tissues (Figures 11A–C), while the expression of MIR600HG was significantly lower in PAAD tissues (Figure 11D). Meanwhile, RT-qPCR revealed that the expression levels of LRRC1, RNF166, and LY6D were increased in PAAD tissues; however, the expression of MIR600HG was decreased in PAAD tissues (Figure 11I). Excitingly, similar results were observed in PAAD cell lines compared to HPDE cell lines (Figures 11E–H).

## Discussion

PAAD is a common lethal cancer with poor prognosis (Wu et al., 2020a). Accurate prediction of prognosis allows for better assessment of patient adaptation to current treatments, including neoadjuvant therapy, surgery and radiotherapy, and immunotherapy. Studies show that TIICs and ceRNA networks are involved in malignancy development, progression, and prognosis (Huang et al., 2019; Zhang et al., 2020). However, little research has been conducted on the construction and the comprehensive analysis of the ceRNA network and TIICs in PAAD to shed light on PAAD treatment, diagnosis, and drug targets. Therefore, the ceRNA network of PAAD, tumor immune infiltration analysis, and drug-related analysis were conducted based on bioinformatics analysis in our study. And a prognostic model of key genes in the ceRNA network and a prognostic model of key TIICs were established, respectively. Meanwhile, we constructed two nomograms to visualize the accuracy of prognostic modules. Combined, LRRC1, MIR600HG, RNF166, M1 macrophages, and CD8 T cells are considered to play key roles in PAAD progression.

MIR600HG is a tumor suppressor in the family of lncRNAs with a protective role in various malignancies. For example, a piece of study on colorectal cancer shows that MIR600HG inhibits colorectal cancer metastasis and increases sensitivity to oxaliplatin chemotherapeutic (Yao and Li, 2020). Another study on oral cancer finds that MIR600HG is positively correlated with the co-expression of ubiquitin-specific proteinase 30, which is a potential therapeutic target as an independent risk factor for oral cancer prognosis (Jiang et al., 2021). Here, as a protective factor of PAAD, the expression of MIR600HG was decreased in PAAD. LRRC1 (leucine-rich repeat-containing protein 1) of the LAP protein family, also known as the LANO protein, contains 524 amino acids (Saito et al., 2001; Li et al., 2013; Lopez Almeida et al., 2018). LRRC1 can regulate downstream signaling through the C-terminal TSV sequence (Yamanaka and Ohno, 2008), the expression of which is higher in hepatocellular carcinoma tissues than in normal liver tissues, to promote tumor proliferation, invasion, and metastasis (Li et al., 2013; Hua et al., 2020). Tumor proliferation, invasion, and metastasis can be inhibited by knocking down LRRC1 to increase the efficacy of ensartinib in advanced lung cancer, as shown in non-small-cell lung cancer studies (Qiu et al., 2021). High expression of LRRC1 is associated with advanced stage and decreased overall survival (OS) in patients with non-small-cell lung cancer. Whole-exome sequencing of 22 patients with familial aggregation of ischemic stroke identified LRRC1 single gene expression as an independent risk factor for stroke development (Ilinca et al., 2020). However, the role of LRRC1 in PAAD has not been investigated. Therefore, our study is novel in reporting the high expression of LRRC1 in PAAD associated with poor prognosis. Ring finger protein 166 (RNF166), a member of the RING finger family, plays a vital role in several physiological activities of cells as ubiquitin ligases (Heath et al., 2016). It has been found that RNF166 determines the recruitment of junctional proteins during antibacterial autophagy and plays a novel pro-apoptotic role in neurotoxin-induced neurodegeneration through XIAP ubiquitination (Oh et al., 2020). Taken together, the correlation between RNF166 and tumor prognosis is first reported in our study.

The infiltration of TIICs in PAAD and the prognostic significance were investigated using the CIBERSORT algorithm. CD8 T cells, M1 macrophages, and activated dendritic cells were deemed independent prognostic factors in PAAD (Masugi et al., 2019). It has been found that the more the CD8^+^ T cells that infiltrate in PAAD, the longer their survival time (Seo et al., 2019). And it was also found that CD8^+^ T cells are fewer in PAAD than in paracancerous tissues and that the combination of PD-1 and CXCR4 results in an increase of CD8^+^ T cells in tumor tissues to promote tumor cell apoptosis. The number of CD8^+^ T cells in the tumor microenvironment in pancreatic and breast cancers is important for response prediction after chemotherapy and immunotherapy (Meyer et al., 2018). M2 macrophages and pro-inflammatory M1 macrophages are associated with the growth and diffusion of tumors and the formation of an immunosuppressive microenvironment (Tsuboi et al., 2019; Qin et al., 2020). The oncogenic role of M1 macrophages in most malignancies has been found. For instance, M1 macrophage infiltration can prolong the survival time of patients with breast, bladder, gastric, osteosarcoma, and colorectal cancers (Xiong et al., 2018; Honkanen et al., 2019; Li et al., 2020a; Zhou et al., 2020). Dendritic cells are the most powerful antigen-presenting cells, and many factors function by activating them (Benkő et al., 2008; Levitz and Golenbock, 2012; Arnold-Schrauf et al., 2015). Meanwhile, it was found that the more the dendritic cell infiltration in gastric cancer tissues, the better the prognosis (Li et al., 2020b). Recent studies show that when gemcitabine is used for the treatment of locally advanced or metastatic pancreatic cancer, the number of naive CD4 T cells and resting memory CD4 T cells slightly increases, which reminds us that these two types of cells are upregulated in gemcitabine-resistant PAAD cells (Ullenhag et al., 2017). This result suggests a potential relationship to the transport and metabolism of gemcitabine associated with the tumor microenvironment of PAAD (Wu et al., 2020b; Kamath et al., 2020).

Previous studies have demonstrated that acquired drug resistance to gemcitabine is a major obstacle of chemotherapy for PAAD (Ding et al., 2020). However, the underlying molecular mechanism of gemcitabine resistance in PAAD has not been fully elucidated (O’Reilly and Abou-Alfa, 2007; Li et al., 2016). Here, we found that the IC50 value of a chemotherapeutic drug for PAAD has no differences in the high-risk and low-risk groups, which means that patients with PAAD easily suffer drug resistance during chemotherapy. Similarly, our research has yielded more consistent results. The AUC value is often calculated with the ROC curve to evaluate prediction models. Because the AUC is the sum of the accuracy and specificity of the calculated model, prediction models with high AUC scores may have high sensitivity but low specificity, which cannot be applied in practice (Vickers and Elkin, 2006; Wu et al., 2020c). However, the DCA curve is more inclined to clinical applicability. Based on the DCA, the superiority of the two prediction models over those of clinical staging and pathology grading was validated in the present study. The risk model constructed by key genes predicts that the AUC values for one-, three-, and five-year OS were 0.709, 0.735, and 0.744, respectively, and the predictive AUC values of the risk model constructed by immune genes for one-, three-, and five-year survival were 0.766, 0.768, and 0.669, respectively, thereby indicating that the risk model can accurately predict the OS of patients with PAAD. We plotted multi-AUC curves to compare the AUC values of the two prediction models at different time points with the AUC values of every single indicator. The above analysis demonstrated the higher prediction accuracy of the model compared to the single indicators and the outperformance of the model in multiple dimensions.

In summary, LRRC1, MIR600HG, RNF166, M1 macrophages, and CD8 T cells are predicted to play significant roles in PAAD. Herein, although we have experimentally validated the data from TCGA and the risk prediction model appears to be relatively stable and practical, it still has some limitations. For instance, further external validation from other databases is also necessary to evaluate the accuracy of the model. Additionally, the mechanism by which MIR600HG regulates downstream targets LRRC1 and RNF166 and affects ICI in PAAD needs to be elucidated experimentally. The prognostic model, nomogram, and drug analysis may provide a certain reference value for pancreatic cancer patient management in the clinic.

## Data Availability

Publicly available datasets were analyzed in this study. The datasets can be found in The Cancer Genome Atlas (TCGA) at https://portal.gdc.cancer.gov and UCSC Xena at https://xenabrowser.net/datapages/.

## Abbreviations

AUC, area under the curve; BP, biological process; CC, cellular component; CI, confidence interval; ceRNA, competitive endogenous RNA; CIBERSORT, cell type identification by estimating relative subsets of RNA transcripts; DCA, decision curve analysis; DEGs, differentially expressed genes; FDR, false discovery rate; FPKM, fragments per kilobase of exon per million mapped fragments; GO, Gene Ontology; HR, hazards ratio; IC50, half-maximal inhibitory concentration; KEGG, Kyoto Encyclopedia of Genes and Genomes; lncRNA, long non-coding RNA; MF, molecular function; miRNA, microRNA; OS, overall survival; PAAD, pancreatic adenocarcinoma; ROC, receiver-operating characteristic; TCGA, The Cancer Genome Atlas; TIDE, Tumor Immune Dysfunction and Exclusion.
